# Insulin-like growth factor 2 mRNA binding protein 3 (IGF2BP3) overexpression in pancreatic ductal adenocarcinoma correlates with poor survival

**DOI:** 10.1186/1471-2407-10-59

**Published:** 2010-02-23

**Authors:** David F Schaeffer, Daniel R Owen, Howard J Lim, Andrew K Buczkowski, Stephen W Chung, Charles H Scudamore, David G Huntsman, Sylvia SW Ng, David A Owen

**Affiliations:** 1 Department of Pathology, The University of British Columbia, Vancouver BC, Canada; 2 Department of Medical Oncology, British Columbia Cancer Agency, Vancouver BC, Canada; 3 Department of Surgery, The University of British Columbia, Vancouver BC, Canada; 4 Department of Advanced Therapeutics, British Columbia Cancer Agency, Vancouver BC, Canada; 5 Faculty of Pharmaceutical Sciences, The University of British Columbia, Vancouver BC, Canada

## Abstract

**Background:**

Pancreatic ductal adenocarcinoma is a lethal disease with a 5-year survival rate of 4% and typically presents in an advanced stage. In this setting, prognostic markers identifying the more agrressive tumors could aid in managment decisions. Insulin-like growth factor 2 mRNA binding protein 3 (IGF2BP3, also known as IMP3 or KOC) is an oncofetal RNA-binding protein that regulates targets such as insulin-like growth factor-2 (IGF-2) and ACTB (beta-actin).

**Methods:**

We evaluated the expression of IGF2BP3 by immunohistochemistry using a tissue microarray of 127 pancreatic ductal adenocarcinomas with tumor grade 1, 2 and 3 according to WHO criteria, and the prognostic value of IGF2BP3 expression.

**Results:**

IGF2BP3 was found to be selectively overexpressed in pancreatic ductal adenocarcinoma tissues but not in benign pancreatic tissues. Nine (38%) patient samples of tumor grade 1 (n = 24) and 27 (44%) of tumor grade 2 (n = 61) showed expression of IGF2BP3. The highest rate of expression was seen in poorly differentiated specimen (grade 3, n = 42) with 26 (62%) positive samples. Overall survival was found to be significantly shorter in patients with IGF2BP3 expressing tumors (P = 0.024; RR 2.3, 95% CI 1.2-4.8).

**Conclusions:**

Our data suggest that IGF2BP3 overexpression identifies a subset of pancreatic ductal adenocarcinomas with an extremely poor outcome and supports the rationale for developing therapies to target the IGF pathway in this cancer.

## Background

Pancreatic ductal adenocarcinoma is a lethal neoplasm with a 5-year survival rate of 4%. Patients typically present with advanced disease. Prognostic markers that identify the more aggressive tumors could aid in management and treatment decisions. The insulin-like growth factor-2 mRNA binding protein family comprises three proteins, IGF2BP1-3, that regulate mRNA transport, translation, and turnover by binding to the coding regions of target mRNAs such as IGF-2 (insulin-like growth factor 2), c-myc, and beta-actin [1-4]. IGF2BP3 was first cloned from a pancreatic tumor cDNA screen and was originally designated as KOC (KH-domain containing protein overexpressed in cancer) [5]. It is known as an oncofetal protein because its expression is highest during embryogenesis [6-10], and is completely silenced in normal adult mouse tissues and is almost so in normal adult human tissues (with fibroblasts, lymphocytes, and the testes being the exceptions) [10,11]. In a previous study, Mueller-Pillasch et al. provided evidence to suggest that IGF2BP3 may play a role in the differentiation of the human exocrine pancreas during embryogenesis [12]. Although the function of IGF2BP3 in pancreatic ductal adenocarcinoma remains unclear, transgenic overexpression of the protein in mice was reported to induce abnormalities in the exocrine pancreas [13]. In addition, recent reports have demonstrated high levels of IGF2BP3 mRNA transcript and protein in pancreatic cancer tissues but not in benign lesions of the pancreas, chronic pancreatitis and/or normal pancreatic tissues [14,15]. The expression of IGF2BP3 has also been associated with an unfavorable outcome in renal clear cell carcinoma [16,17] and more recently, in ovarian clear cell carcinoma [18]. Collectively, these observations led us to postulate that IGF2BP3 expression could be a prognostic indicator for pancreatic ductal adenocarcinoma. The objective of the present study was to determine if expression of IGF2BP3 correlates with patient prognosis.

## Methods

### Patients and tumor specimens

The expression of IGF2BP3 and IGF-2 was evaluated by immunohistochemistry on a tissue microarray of 127 pancreatic adenocarcinoma, ranging from tumor grade 1, 2 and 3, (according to WHO criteria [19]), from patients who underwent surgical resection at Vancouver General Hospital. A retrospective analysis of the available clinical data was performed. Ethics approval was obtained from the University of British Columbia Ethics Review Board. Age, gender, concomitant illnesses, previous surgeries, and survival data, was collected via a retrospective chart review of the patients referred to the British Columbia Cancer Agency and transferred to an anonymized database. Pathological staging and histological tumor grade were determined from the original hematoxylin and eosin stained slides. According to the AJCC (6^th ^edition) classification, the study cohort was distributed as follows: IA (n = 1), IB (n = 34), IIA (n = 19), IIB (n = 65), III (n = 6) and IV (n = 2). Resection status was determined as follows: R0 (n = 82), R1 (n = 37) and R2 (n = 8). Clinical and morphological data are summarized in Table 1.

**Table 1:** Clinicopathological characteristics by tumor grade

	N	Gender		Age	Follow-up	Treatment
Cohort by Grade		M	F	Mean ± SD	[month]	Adjuvant Chemotherapy
WHO 1	24	18	6	60.8 ± 11.5	19 ± 3.6	6 (25%)
WHO 2	62	44	18	57.4 ± 12.3	13 ± 6.1	16 (26%)
WHO 3	42	30	12	54.3 ± 14.2	7 ± 4.8	28 (66%)
						
Sum/*Mean*	128	92	36	*57.5 ± 12.6*	*13 ± 4.8*	*50 (39%)*

SD - standard deviation

A tissue microarray (TMA) was constructed using duplicate 0.6-mm cores generated from representative areas of formalin-fixed, paraffin-embedded surgical excision blocks that had been reviewed by at least two pathologists with appropriate subspecialty expertise. Non-neoplastic pancreatic parenchyma (n = 14) served as control tissue.

### Treatment and outcome

The majority of patients with ductal adenocarcinoma received surgical treatment with curative intent and did not receive adjuvant chemotherapy. If given, adjuvant chemotherapy was heterogeneous and included either 5FU alone, gemcitabine, or a combination of 5FU with cisplatin. At the time of data collection, generalized province wide treatment guidelines for this particular neoplasm were still in development. The study endpoint was defined as disease specific survival. This information was available for 127 patients. Mean follow-up time was 13 ± 4.8 months (Table 1).

### Immunohistochemical staining and scoring

Four-micron sections from the arrays were stained with hematoxylin and eosin to confirm the presence of representative tumor in each core. Sections were stained using a mouse monoclonal antibody against IGF2BP3 (clone 69.1, 1:100 dilution, DAKO, Carpenteria, CA) [20] and an anti-IGF-2 rabbit polyclonal antibody (1:100 dilution, Abcam, Cambridge, MA) raised against a recombinant human IGF-2 protein. This specific IGF-2 antibody recognizes both the prohormone form of IGF-2 (also named 'big IGF-2, 15 kDa') and the smaller IGF-2 (7.5 kDa). Immunostaining was performed on a Ventana Discovery XT (Ventana, Tucson, AZ) using a standard CC1 heat-induced epitope retrieval protocol (Ventana, Tucson, AZ), and DABMap detection system (Ventana, Tucson, AZ). A cervical carcinoma was used as a positive control with every staining run, and normal pancreatic tissue, which was non-immunoreactive, was used as a negative control. To aid the analysis of the numerous tissue cores stained by immunohistochemistry, digital images were collected using a BLISS instrument (Bacus Laboratories, Lombard, IL). Tissue cores were scored on the basis of thepercentage of positive tumor cells staining above background intensity in a membranous and/or cytoplasmic pattern according to published methodology [21]. In tumor cells, the staining intensity was designated as either non-existent (0), weak (1), moderate (2) or strong (3). The number of cells was scored as either no cells stained (0), <10% (1), 10-50% (2), 50-80% (3) or > 80% (4). The final score was calculated by multiplying these two variables. A score of 0-5 was considered negative (IGF2BP3 and IGF-2 not overexpressed), and a score of 6 and higher was considered positive (overexpression of IGF2BP3 and IGF-2) in accordance with Koebel et al. [21]. Discrepant score results for duplicate cores, when present, were consolidated as the higher interpretable score [22]. Cut-off point for positive cases was any convincing cytoplasmic expression in more than 5% of tumor cells. IGF2BP3 and IGF-2 staining on the tissue microarray was scored by two pathologists (DFS and DRO) blinded to clinical outcome. Discordant results were reviewed by a senior pathologist (DAO) and consensus was reached.

### Statistical Analysis

SPSS for Windows (Chicago, IL, USA; Version 14.0) was used for statistical analyses. Survival curves were plotted using the Kaplan-Meier method, with significance assessed using log-rank tests. Cox proportional hazard model was applied to perform multivariate analysis to determine the independent effects of IGF2BP3 expression, age, AJCC stage group, lymphovascular invasion, histological grade and perineural invasion. Statistical significance was declared if the P-value was < 0.05.

## Results

### IGF2BP3 expression pattern and survival

**Figure 1:**
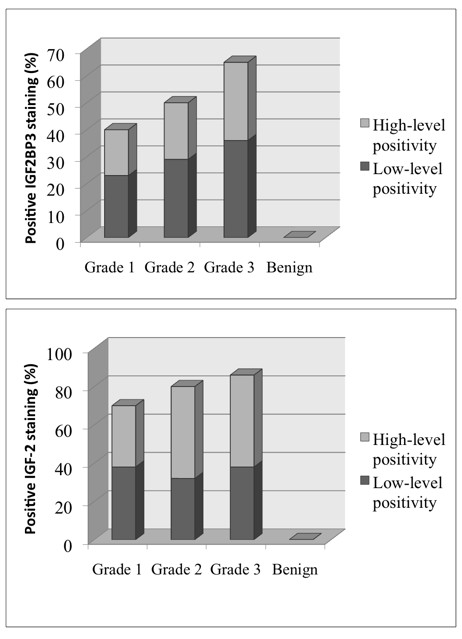
**Expression of IGF2BP3 and IGF-2 staining**. Bar graphs showing the percentages of low and high level positive (overexpression) staining for IGF2BP3 and IGF-2 of grade 1, 2, and 3 pancreatic ductal adenocarcinomata, compared to benign ductal pancreatic tissues. Data was obtained from 127 pancreatic ductal adenocarcinoma cases and 14 benign cases.

**Figure 2:**
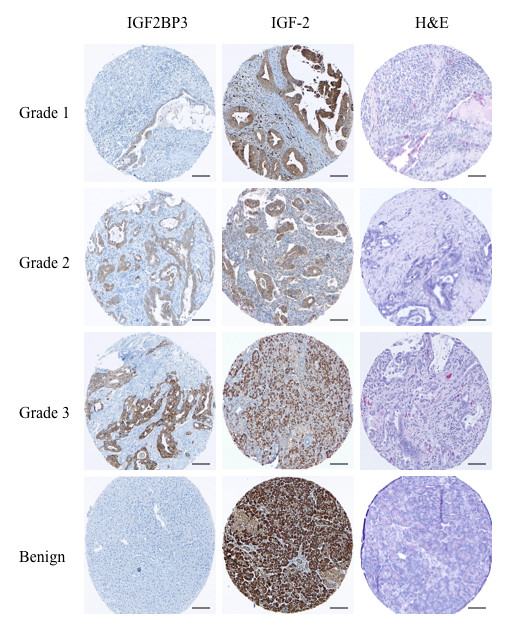
**Immunohistochemical staining for IGF2BP3 and IGF-2 by tumor grade**. Representative tissue microarray cores of pancreatic ductal adenocarcinomata with immunohistochemical staining for IGF2BP3 and IGF-2 by tumor grade. Note the complete lack of immunohistochemical staining for IGF2BP3 in benign pancreatic tissue. While there appears to be positive staining for IGF-2 in the benign cores, this was exclusively acinar staining and not ductal. [Scale bar, 100 μm.]

**Figure 3:**
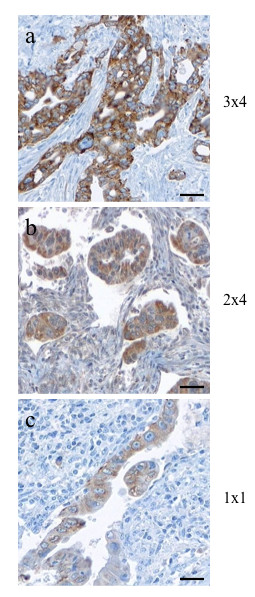
**Evaluation of staining intensity and number of stained cells**. IGF2BP3 (a) and IGF-2 (b) with strong and moderate staining intensity in more than 80% of pancreatic ductal adenocarcinoma cells, given a score of 12 and 8, respectively. In comparison weak staining of IGF2BP3 (c) in less than 10% of cells in an example of a grade 1 tumor, given a score of 1. [Scale bar, 200 μm.]

IGF2BP3 is selectively overexpressed in pancreatic ductal adenocarcinoma tissues but not in benign pancreatic tissues, consistent with previous reports [14,15]. In our cohort, 63% of ductal adenocarcinoma showed IGF2BP3 expression (Figure 1). IGF2BP3 expression differed between tumor grade (P < 0.001, Pearson's Chi-square). The highest rate of expression was seen in poorly differentiated specimens (grade 3, n = 42) with 26 (62%) positive samples, followed by grade 2 (44%, n = 27), and with the lowest rate of expression in tumour grade 1 samples (38%, n = 9). Figure 2 shows representative H&E and IGF2BP3 staining of the tissue microarray. The staining was readily appreciable in the tumor cells as shown in a higher magnification photomicrograph (Figure 3a). While IGF2BP3 expression was also observed in premalignant pancreatic intraepithelial neoplasia (PanIN) lesions, mainly PanIN-3, this staining did not show evidence of a grade dependent expression.

**Figure 4:**
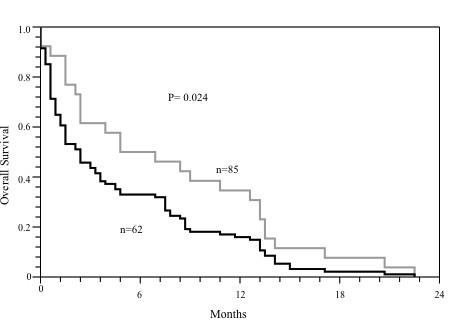
**Disease specific Survival Analysis**. Kaplan-Meier analysis of disease specific survival in pancreatic ductal adenocarcinoma expressing IGF2BP3 (black line, n = 62) and not expressing IGF2BP3 (grey line, n = 65). P value was calculated using the log-rank test.

Disease specific survival for all tumor grades was found to be significantly shorter in patients with IGF2BP3 expressing tumors than in those with IGF2BP3 non-expressing tumors (9.3 months vs. 13.7 months, respectively, P = 0.024, Figure 4). For IGF2BP3 expression, a risk ratio of 2.3 (95% CI 1.2-4.8), independent of grade, was calculated and when stratified by treatment subgroup, IGF2BP3 expression showed the same trend for unfavorable prognosis.

To determine and compare the individual risk of IGF2BP3 expression, age, AJCC stage group, lymphovascular invasion, histological grade and perineural invasion, a Cox proportional hazard analysis was performed (Table 2). IGF2BP3 was found to be a significant independent predictor of risk for the overall survival by increasing the risk of death (HR 3.34 ± 1.93; P = 0.05). However, no statistical significant difference was found in comparing IGF2BP3 expression versus lack of expression in regard of resection status or administration of chemotherapy.

**Table 2:** Multivariable Cox regression analyses

	Hazard ratio	SE	*p*-value	95% CI
IGF2BP3	3.343	1.931	0.051	1.921 - 8.963
Age	1.032	0.017	0.673	1.002 - 1.0681
AJCC				
IA	2.21e - 13	4.38e - 06	1.000	0
IB	4.377	4.292	0.126	0.661 - 28.991
IIA	2.496	1.754	0.226	0.571 - 10.873
IIB	2.152	1.413	0.257	0.096 - 3.462
III	2.564	2.034	0.248	0.523 - 7.639
IV	1.76e - 09	3.84e-07	1.000	0
LVI	1.876	0.862	0.506	0.203-5.476
WHO				
1	0.704	0.431	0.563	0.214-2.335
2	0.651	0.429	0.510	0.187-2.371
3	0.582	0.527	0.554	0.099-3.627
PNI	1.456	0.873	0.738	0.411-3.452

SE - standard error, LVI - lymphovascular invasion, PNI - perineural invasion

### IGF-2 expression pattern and its correlation with IGF2BP3 expression

Since the most studied IGF2BP3-regulated mRNA transcript is IGF-2, we examined the protein expression of IGF-2 in relation to that of IGF2BP3. IGF-2, in comparison to IGF2BP3, was expressed not only in pancreatic ductal adenocarcinoma, but also in benign pancreatic tissue (Figure 2). However, the staining within benign tissue was exclusively located in acinar cells and, like IGF2BP3, absent in ductal cells, implying IGF2BP3-independent regulation of IGF-2 expression in the acinar cells. If present, the intensity of IGF-2 positive staining was almost 2-fold higher than that of IGF2BP3 and readily observable (Figure 2 and Figure 3b, c). While the expression of IGF-2 was slightly different between tumor grades, this was not statistical significant (P = 0.093; Figure 1). The expression of IGF2BP3 was significantly and positively correlated with that of IGF-2 in tumor cells (P = 0.02). Calculations for disease specific survival for IGF-2 expression alone did not show any difference between the cohorts (data not shown).

## Discussion and Conclusions

Although IGF2BP3 is expressed in a variety of malignant neoplasms including pulmonary small cell [23], endometrial [20], and cervical carcinomas [24], the prognostic value has been demonstrated only in renal clear cell carcinomas [16,17], low-stage urothelial carcinomas of the bladder [25], and more recently in ovarian clear cell carcinoma [18]. The present study showed for the first time that IGF2BP3 overexpression correlates with poor survival in pancreatic ductal adenocarcinoma. We also demonstrated that the expression of IGF-2 significantly correlates with that of IGF2BP3, which has been previously reported to promote IGF-2 mRNA translation [2]. An attractive feature of IGF2BP3 as a biomarker in pancreatic ductal adenocarcinoma is that its expression is only found in tumor tissue and is absent in normal adult pancreatic ductal tissue. This on-off pattern of expression makes staining interpretation very simple in practice. The expression of IGF2BP3 during embryogenesis [12] but not in adulthood suggests that IGF2BP3 is epigenetically silenced in adult tissues. In pancreatic ductal adenocarcinoma, re-expression of IGF2BP3 might be the result of promoter hypomethylation. The IGF2BP3 gene is located on chromosome 7p (at location 23,316,354-23,476,520), a region not subject to frequent perturbation in pancreatic ductal adenocarcinoma [26]. It is therefore unlikely that gene amplification is responsible for the observed IGF2BP3 expression in pancreatic ductal adenocarcinoma. We are currently testing the hypothesis that the IGF2BP3 promoter is hypomethylated in pancreatic ductal adenocarcinoma, and that this correlates with expression levels. If this hypothesis holds true, IGF2BP3 could be regarded as a target for re-methylating enzymes.

The mechanisms by which IGF2BP3 and IGF-2 facilitate tumor progression in pancreatic ductal adenocarcinoma remain to be elucidated. However, IGF2BP3-mediated activation of IGF-2 translation has been shown to increase human leukemia cell proliferation [2]. Vikesaa et al. [27] reported that IGF2BP3 modulates the expression of specific extracellular matrix and cell adhesion proteins (e.g., collagen V α1, ALCAM) and stabilizes CD44 mRNA, thereby promoting invadopodia formation in cervical cancer cells. Moreover, IGF2BP3 has been demonstrated to enhance the motility of human colorectal cancer cells [28]. While less studied than its family member IGF-1, IGF-2 is also known to play a role in cancer progression. Corcoran et al. [29] recently noted that induction of IGF-2 expression in pre-malignant lesions coincides with progression to advanced medulloblastoma. In addition to its direct effect on tumor cells, IGF-2 was shown to promote tumor angiogenesis and lymphangiogenesis [30,31]. Decreases in IGF-2 mRNA levels and IGF-2 secretion using growth hormone-releasing hormone antagonists were associated with decreased cancer cell proliferation *in vitro *[32] and tumor growth *in vivo *[33,34]. Furthermore, IGF-2 deficient tumor cells were shown to be more sensitive to chemotherapy-induced apoptosis [35]. It seems reasonable to speculate that the IGF2BP3/IGF-2 pathway may drive progression in pancreatic ductal adenocarcinoma by similar means, and that therapeutically targeting either or both of these proteins may result in tumor control. These possibilities are being investigated in our laboratory. Interestingly, Wang et al. reported that IGF2BP3 is immunogenic in lung cancer patients, suggesting that it may be a potential target for immunotherapy [36]. In a recent Phase I trial, IGF2BP3 vaccine was shown to be safe and well tolerated [37].

In summary, this is a retrospective study of a post-surgical, heterogeneously treated cohort of patients with resected pancreatic adenocarcinoma. Thus, prognostic associations, defined as tumor behavior after primary surgery and uninfluenced by different regimens of adjuvant therapy, cannot be assessed. A strength of this study is the relatively large sample size (n = 127) of pancreatic ductal adenocarcinoma. Our data suggest that IGF2BP3 overexpression denotes a subset of pancreatic adenocarcinomas with an extremely poor outcome, and supports the rationale for developing therapies to target the IGF2BP3/IGF-2 pathway in pancreatic ductal adenocarcinoma.

## Competing interests

The authors declare that they have no competing interests.

## Authors' contributions

DFS designed the study, carried out the immunohistochemistry scoring, performed the statistical analysis and drafted the manuscript. DRO participated in the immunohistochemistry scoring and collected patient data. HJL participated in patient data collection and statistical analysis. AKB, SWC and CHS participated in tissue procuring and the design of the study. DGH, SSWN and DAO contributed to the design and coordination of the study, manuscript preparation and served as principal supervisors. All authors read and approved the final manuscript.

## Pre-publication history

The pre-publication history for this paper can be accessed here:

http://www.biomedcentral.com/1471-2407/10/59/prepub
